# A triple action CDK4/6-PI3K-BET inhibitor with augmented cancer cell cytotoxicity

**DOI:** 10.1038/s41421-020-0181-z

**Published:** 2020-07-28

**Authors:** Adam M. Burgoyne, Kendra R. Vann, Shweta Joshi, Guillermo A. Morales, Francisco M. Vega, Alok Singh, Dhananjaya Pal, Aran B. Merati, Tatiana G. Kutateladze, Donald L. Durden

**Affiliations:** 1 Division of Hematology-Oncology, Department of Medicine, Moores Cancer Center, University of California San Diego, La Jolla, CA USA; 2 Department of Pharmacology, University of Colorado School of Medicine, Aurora, CO USA; 3 Division of Pediatric Hematology and Oncology, Department of Pediatrics, Moores Cancer Center, University of California San Diego, La Jolla, CA USA; 4 SignalRx Pharmaceuticals, San Diego, CA USA; 5 Department of Cell Biology, Instituto de Biomedicina de Sevilla, Universidad de Sevilla, Sevilla, Spain

**Keywords:** X-ray crystallography, Epigenetics, Cancer therapy

Dear Editor,

The PI3K-AKT-mTOR pathway has been at the center of anti-cancer drug development^1,2^, but targeted inhibition of PI3K kinase activity is proven to provide a limited therapeutic effect and is often followed by the development of resistance to the drug^3^. This limitation could in part be alleviated through concurrently inhibiting multiple pathological pathways characterized by synthetic lethality relationship. For example, beneficial effects in metastatic breast cancer are observed when inhibition of PI3K is combined with BET proteins inhibition^4^. While the need for combinations of single agent targeted therapies has become clear, this approach commonly requires dose reduction of the individual agents due to additive toxicity that may compromise efficacy^5^.

One strategy being explored to overcome the drawbacks of combination-based targeted therapy relies on the development of single-molecule inhibitors highly specific toward several targets. We and others have previously reported potent dual activity inhibitors, including PI3K-BET inhibitors^6,7^, cyclin-dependent kinases (CDK)-BET and mitogen-activated protein kinases (MAPK)-BET inhibitors^8^, and CDK-PI3K inhibitors^9^. In this study, we describe the first in class rationally-designed triple activity inhibitor that concomitantly disrupts functions of three critical targets based upon known synthetic lethality relationships in cancer cells—CDK4/6, PI3K, and BRD4. The triple inhibitor SRX3177 has broad cytotoxic activity against a variety of tumor types. Our results using a cyclin D1-dependent hematologic malignancy, a MYC-dependent embryonal tumor, and a PI3K-dependent solid tumor demonstrate that SRX3177 is efficacious and non-toxic in vitro to normal epithelial cells.

We originally generated a series of chemical probes that simultaneously bind CDK4/6, PI3K, and BRD4 based on analyses of the crystal structures of the target domains and in silico modeling. By screening these compounds against the three targets using in vitro binding assays, kinase assays, and CDK assays, we identified the most potent compound, SRX3177 (Fig. 1a). SRX3177 showed nanomolar potency against PI3Kα, both bromodomains of BRD4 (BD1 and BD2), and CDK4/6 (Fig. 1b). The specificity of SRX3177 and lack of off-targeting was confirmed by KINOME*scan* and BROMO*scan* assays (Fig. 1c, d and Supplementary Table S1).

**Fig. 1:**
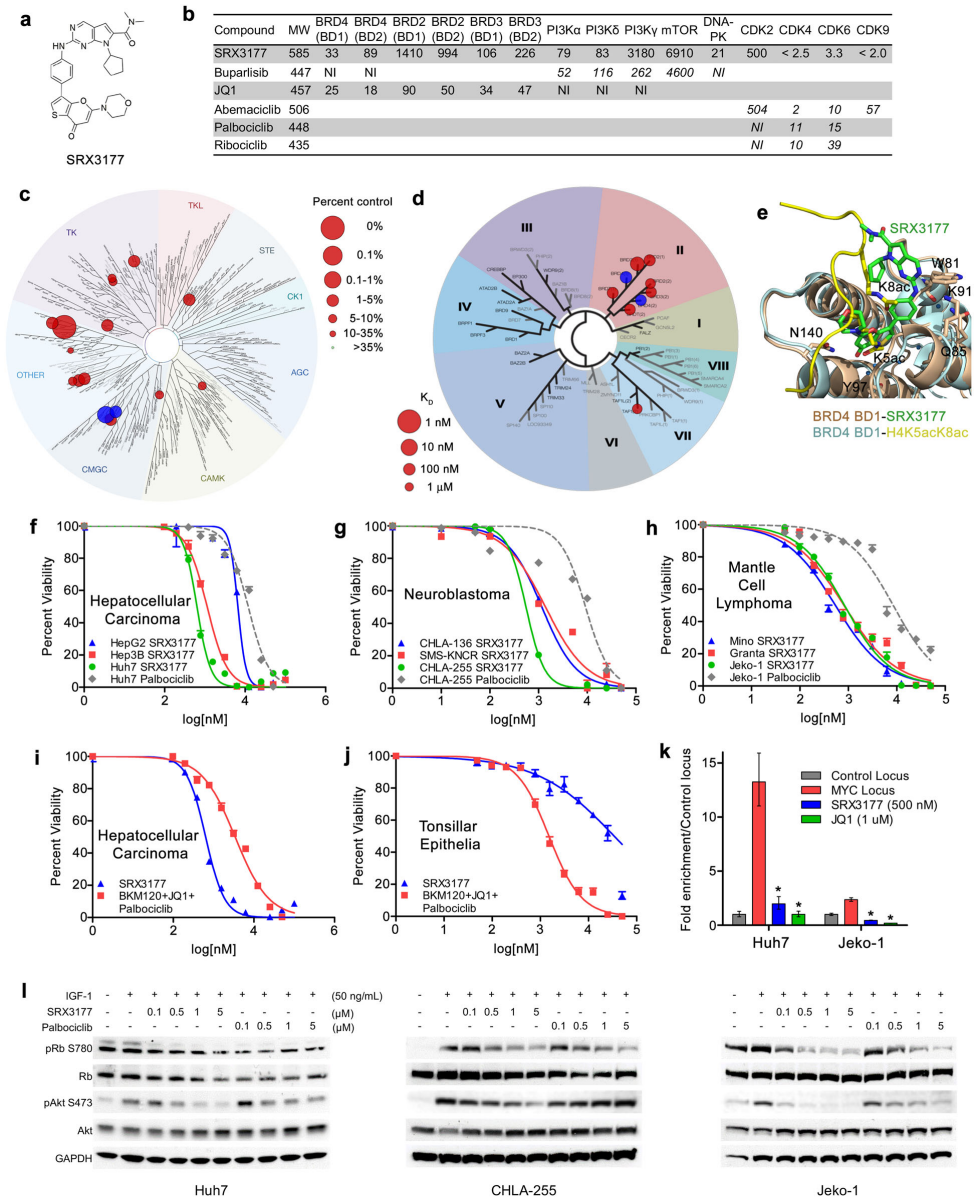
SRX3177 is a novel triple action inhibitor. **a** Structure of SRX3177. **b** In vitro bromodomain binding and kinase activities were performed to measure IC_50_ values which are displayed as nM concentrations. Italicized values were obtained from the literature. **c**, **d** KINOME*scan* (**c**) and BROMO*scan* (**d**) data were analyzed using the TREE*spot*. Target effects are indicated by red circles. **e** Structural basis for the recognition of SRX3177 by BRD4 BD1. Overlay of the structures of BRD4 BD1 (light brown) in complex with SRX3177 (green) and H4K5acK8ac peptide (PDB: 3UVW) (yellow). **f**–**h** Hepatocellular carcinoma (HepG3, Hep3B, and Huh7), neuroblastoma (CHLA-136, SMS-KNCR, and CHLA-255), and mantle cell lymphoma (Mino, Granta-519, and Jeko-1) were treated with increasing doses of SRX3177 or palbociclib and assessed for viability for determination of IC_50_. **i**, **j** Huh7 and normal tonsillar epithelial cells were treated with SRX3177 versus combination of BKM120, JQ1, and palbociclib and assessed for viability and IC_50._**k** ChIP assays using Huh7 and Jeko-1 cells treated with SRX3177. Purified immunoprecipitated DNA and input DNA was amplified by quantitative real-time PCR and analyzed for enriched binding of BRD4 to the *MYC* transcription start site in comparison with binding to a nonspecific locus upstream of *MYC*. Data were normalized and plotted as percentage of vehicle control binding. Asterisks denote *p* ≤ 0.05 in comparison with vehicle control by unpaired Student’s *t*-test. **l** Serum starved Huh7, CHLA-255, and Jeko-1 cells were stimulated with 50 ng/mL IGF-1 and treated with SRX3177 or palbociclib. Lysates were immunoblotted for pRb, Rb, pAkt, Akt, and GAPDH.

To gain insight into the molecular basis for inhibition of BRD4, we assessed binding of SRX3177 to BD1 and BD2 of BRD4 using NMR and X-ray crystallographic approaches. Large chemical shift perturbations in ^1^H,^15^N heteronuclear single quantum coherence (HSQC) spectra of ^15^N-labeled BRD4 BD1 and BD2 were observed upon gradual addition of SRX3177, demonstrating that SRX3177 directly targets both bromodomains of BRD4 (Supplementary Fig. S1). A slow-to-intermediate exchange regime on the NMR chemical shift time scale indicated a tight interaction, in full agreement with the nanomolar values of K_d_ and IC_50_.

To obtain the atomic-resolution mechanistic details, we co-crystallized BRD4 BD1 with SRX3177 and determined the crystal structure of the BD1-SRX3177 complex (Supplementary Table S2). In the complex, SRX3177 occupies a large hydrophobic pocket located at one of the open ends of the BD1 four-helix bundle (Fig. 1e and Supplementary Fig. S2a, b). The base of the SRX3177-binding pocket is lined with a well-defined water shell, which is conserved in other BRD4 BD1 complexes^10^. Superimposition of the SRX3177-bound and H4K5acK8ac-bound BRD4 BD1 structures reveals that SRX3177 acts as an acetyllysine mimetic. Particularly, a hallmark contact, which is required for functional bromodomains, involves formation of the hydrogen bond between the acetyllysine substrate and the side chain of Asn140. In the BD1-SRX3177 complex, the side chain of Asn140 is hydrogen bonded to the thienopyrano group of SRX3177 (Supplementary Fig. S2c).

We then tested the efficacy of SRX3177 in the NCI-60 Panel of Human Tumor Cell Line Screen at the National Cancer Institute, in which the triple inhibitor SRX3177 had cytotoxic effects on the majority of the cell lines tested, with activity against cells derived from all underlying tumor histologies (Supplementary Fig. S3). SRX3177 displayed greater tumor cytotoxicity as compared to the dual BRD4/PI3K inhibitor, SF2523 or dual BRD4/CDK4/6 inhibitor, SRX3177P (Supplementary Fig. S3). To characterize the effect of SRX3177 in detail, we measured cytotoxic activity of this inhibitor in a cyclin D1-dependent hematologic malignancy (mantle cell lymphoma), a MYC-dependent embryonal tumor (neuroblastoma), and a PI3K-dependent solid tumor (hepatocellular carcinoma) (Fig. 1f–h). The dose-response curves generated from cell-based cytotoxicity assays in panels of cell lines from each of these tumor types revealed a strong antitumor activity of SRX3177, with IC_50_ values in the nanomolar range (Fig. 1f–h and Supplementary Table S3). Furthermore, SRX3177 was ~10–20-fold more potent than the CDK4/6 inhibitor palbociclib in these tumor cell lines.

To further evaluate the anticancer activity of SRX3177, we compared the cytotoxic effects caused by SRX3177 to the cytotoxic effects caused by a combination of individual PI3K, BRD4, and CDK4/6 inhibitors of similar potency (Fig. 1i, j). Huh7 cells were treated with either SRX3177 or the combination of BKM120 (buparlisib), JQ1, and palbociclib and assessed for cytotoxicity. Equimolar combination of the three drugs had a combined IC_50_ of 3.4 µM, which is 5-fold less potent than that of SRX3177 (Fig. 1i). Furthermore, when normal tonsillar epithelial cells were treated with either SRX3177 or the combination of BKM120, JQ1, and palbociclib (as a surrogate for toxicity of normal tissue), the toxicity was increased 20-fold (Fig. 1j). Altogether, these data suggest that the triple inhibitor SRX3177 has higher efficacy and is substantially less toxic to normal cells in vitro in comparison with the combination of individual CDK4/6, PI3K and BRD4 inhibitors of similar potency.

To examine the effect of SRX3177 on BRD4 chromatin binding function, we measured occupancy of BRD4 at the transcriptional start site (TSS) of *MYC*, a known target of BRD4, by chromatin immunoprecipitation (ChIP). Treatment of Huh7 and Jeko-1 cells with SRX3177 abrogated binding of BRD4 to the *MYC* TSS, indicating that SRX3177 has pharmacodynamic activity against its BRD4 target in cell-based models (Fig. 1k). Finally, immunoblotting of lysates from Huh7, CHLA-255, and Jeko-1 cells stimulated with IGF-1 and treated with increasing concentrations of SRX3177 showed a decrease in levels of Rb phosphorylation (as a downstream surrogate of CDK4/6 activity) and Akt phosphorylation (as a downstream surrogate of PI3K activity) (Fig. 1l). Thus, SRX3177 effectively inhibits its targets BRD4, CDK4/6, and PI3K in cell-based assays.

In conclusion, in this study we report the first triple action single-molecule inhibitor SRX3177, which disrupts cancer cell signaling through simultaneously inhibiting CDK4/6, PI3K, and BRD4. SRX3177 displays excellent kinome and BET bromodomain selectivity and marked cytotoxicity across multiple tumor types. Our results demonstrate that SRX3177 is efficacious and non-toxic to normal cells in vitro in a wide range of tumor models suggesting further evaluation of this distinct chemotype in resistant cancer models.

## Supplementary information

Supplementary Information

Supplementary Table S1

Validation report

## Data Availability

The atomic coordinates and structure factors have been deposited in the Protein Data Bank under accession code 6WW8. All other relevant data supporting the key findings of this study are available within the article and its Supplementary information files or from the corresponding authors upon reasonable request.

## Supplementary information

**Supplementary Information** accompanies the paper at (10.1038/s41421-020-0181-z).
