# Supplementary material for: A triple action CDK4/6-PI3K-BET inhibitor with augmented cancer cell cytotoxicity
Source: Cell Discov. 2020 Jul 28;6:49. doi: 10.1038/s41421-020-0181-z (PMC7385619; doi:10.1038/s41421-020-0181-z)
Supplement: Supplementary file 1 — Supplementary Information [file 41421_2020_181_MOESM1_ESM.pdf]

Supplementary Information

A triple action CDK4/6-PI3K-BET inhibitor with augmented cancer cytotoxicity

Adam M. Burgoyne, Kendra R. Vann et al.,

Methods

Cell lines and reagents

The human hepatocellular carcinoma cell lines Hep3B and HepG2 were obtained from American Type Culture Collection (ATCC, Manassas, VA); the Huh7 cell line was obtained from the Japanese Collection of Research Bioresources Cell Bank (Tokyo, Japan). Hep3B, HepG2, and Huh7 cells were grown in high-glucose Dulbecco's Modification of Eagle's Medium with 4 mM L-glutamine (DMEM, Corning, Manassas, VA) supplemented with 10% fetal bovine serum (FBS, VWR, Radnor, PA), 1 mM sodium pyruvate (ThermoFisher Scientific, Waltham, MA), and 1X antibiotic-antimycotic solution (Corning). Human neuroblastoma cell lines CHLA-255, CHLA-136, and SMS-KNCR were a gift from Dr. Robert Seeger (Children's Hospital Los Angeles, Los Angeles, CA) and were grown in Iscove's Modified Dulbecco's Medium with 4 mM L-glutamine and 25 mM HEPES (IMDM, ThermoFisher Scientific) supplemented with 10% FBS and 1X antibiotic-antimycotic solution. Human mantle cell lymphoma cell lines Granta-519, Mino, and Jeko-1 were a gift from Dr. Thomas Kipps (UC San Diego, La Jolla, CA). Granta-519 cells were maintained in DMEM with 4 mM L-glutamine supplemented with 20% FBS and 1X antibiotic-antimycotic solution; Mino and Jeko-1 cells were maintained in RPMI 1640 with 2 mM L-glutamine supplemented with 10% FBS (Jeko-1) or 20% FBS (Mino) and 1X antibiotic-antimycotic solution. Normal human tonsillar epithelial cells (RRP008) were obtained from the biorepository at Rady Children's Hospital (San Diego, CA) under an Institutional Review Board-approved protocol. Cells were maintained in EpiLife® Medium (ThemoFisher Scientific) supplemented with 40 µg/mL bovine pituitary extract (ThermoFisher Scientific), 10 ng/mL recombinant human epidermal growth factor (ThermoFisher Scientific), 10ng/mL recombinant human fibroblast growth factor (R&D Systems, Minneapolis, MN), Y-27632 ROCK inhibitor (Enzo Life Sciences, Farmingdale, NY), and 1X antibiotic-antimycotic solution.All cells were authenticated by short tandem repeat DNA profiling at the respective cell banks and were maintained at 37 °C in a 5% CO_2 atmosphere as recommended by the suppliers. JQ1 was a gift

from James Bradner (Dana-Farber Cancer Institute, Boston, MA). BKM120 (buparlisib) was from Novartis (Basel, Switzerland). Palbociclib was obtained from LC Laboratories (Woburn, MA). Recombinant human IGF-1 was from Peprotech (Rocky Hill, NJ). Antibodies specific for AKT (#9272), pAKT Ser473 (#9271), Rb (#9309), and pRb Ser780 (#9307) were obtained from Cell Signaling Technology (Beverly, MA). GAPDH antibody (GT239) was from GeneTex, Inc. (Irvine, CA).

In silico modeling

Molecular models were created to design and dock molecules *in silico* using the software engines AutoDock Vina[1] and rDock[2] and the crystal structures of BRD4 (PDB ID: 4CFK), PI3K (PDB ID: 4JPS) and CDK6 (PDB ID: 5L2T).

Bromodomain and kinase assays

Inhibition of BET family members was measured using Alpha Screen assays from Reaction Biology (Malvern, PA) with His-tagged bromodomains and acetylated histone peptides as ligands. CDK and PI3K isoform activities were measured using *in vitro* kinase assays from Reaction Biology and ThermoFisher Scientific (Waltham, MA), respectively. KINOME*scan* and BROMO*scan* assays were performed at Eurofins DiscoverX (Fremont, CA) with data analyzed using the TREE*spot* tool.

Protein expression and purification

BRD4 BD1 (aa 43-180) and BD2 (aa 342-460) were expressed in *E. coli* BL21 (DE3) RIL in either Luria Broth or M19 minimal media supplemented with $^{15}NH_4Cl$. Cells were harvested by centrifugation and resuspended in 50 mM HEPES (pH 7.5) supplemented with 150 mM NaCl and 1 mM DTT. Cells were lysed by freeze-thaw followed by sonication. Uniformly ^{15}N-labeled

and unlabeled proteins were purified on glutathione Sepharose 4B beads and the GST tag was cleaved with PreScission or thrombin proteases, essentially as described[3].

Crystallization and structure determination of BRD4 BD1-SRX3177 complex

The FPLC-purified BRD4 BD1 (aa 43-180) protein was dialyzed against 10 mM HEPES pH 7.5, 150 mM NaCl, 1 mM TCEP with a 2 M equivalence of the SRX3177 compound at 4 °C for 48 hours, then concentrated to 9 mg/mL. Crystals were grown at 18 °C using a sitting-drop vapor diffusion method against 100 mM Tris pH 8.5, 25% (w/v) PEG 3350, and 200 mM NH_4Cl. The 1.6 μL crystallization drop contained a 1:1 ratio of protein-ligand:reservoir solution. Co-crystals of BRD4 BD1 with SRX3177 were cryoprotected with 30% (w/v) glycerol. X-ray diffraction data were collected at 100 K on a Rigaku Micromax 007 High Frequency rotating anode generator with a Pilatus 200K Hybrid Pixel Array Detector. The dataset was indexed and scaled using HKL3000[4], and phases were determined by molecular replacement with Phaser[5] using a modified version of PDB:3MXF as a search model. Refinement of the model was carried out using PHENIX Refine[6] and manually in Coot[7].

NMR spectroscopy

NMR spectra were recorded at 25 °C on a Varian INOVA 600 MHz spectrometer outfitted with a cryogenic probe. Two dimensional ^{1}H,^{15}N heteronuclear single quantum coherence (HSQC) experiments were collected on the uniformly ^{15}N-labeled BRD4 BD1 and BD2 in PBS buffer pH 6.8, 8% D_2O as SRX3177, resuspended in DMSO, was gradually added.

NCI-60 Human tumor cell lines screen

SRX3177, SRX3177P, and SF2523 were analyzed in the NCI-60 Human Tumor Cell Lines Screen developed in the Developmental Therapeutics Program at the National Cancer Institute. Cancer cell lines were treated with investigational drugs at 10 μM per protocol for One-dose

assay for comparison relative to the no-drug control and to the time zero number of cells for assessment of growth inhibition (values between 0 and 100) and lethality (values less than 0), respectively.

Cell viability assays

Hepatocellular carcinoma cells (Hep3B, HepG2, and Huh7), neuroblastoma cells (CHLA-255, CHLA-136, and SMS-KNCR), mantle cell lymphoma (Granta-519, Mino, and Jeko-1), or normal tonsillar epithelial cells (RRP008) were seeded in complete medium in 96-well tissue culture plates at 2.5 x 10^3 to 1 x 10^4 cells per well, respectively. Cells were incubated overnight to allow for attachment of adherent cells. SRX3177 was added to cells in serial 1:2 dilution starting at 50 or 100 µM in triplicate the following day. Cells were incubated for 48 hours at 37 °C then treated with either CCK8 reagent (Dojindo Molecular Technologies, Rockville, MD) or Alamar Blue (ThermoFisher Scientific) and incubated for 3-6 hours at 37 °C. For CCK8, absorbance was measured at 450 nm; for Alamar Blue, fluorescence signals were read as emission at 590 nm after excitation at 560 nm. Averaged values were normalized as a percentage of control and plotted and regressed for dose response using GraphPad Prism (GraphPad Software, Inc., La Jolla, CA).

Chromatin immunoprecipitation

Chromatin immunoprecipitation assays using antibodies against BRD4 and an isotype control and primers designed to the *MYC* transcriptional site and a non-regulated upstream locus were performed as previously described[8].

Immunoblotting

For immunoblotting experiments, adherent or suspension cells were growth arrested by overnight incubation in basal medium without FBS and then stimulated with IGF-1 and/or

incubated with drug of interest for 30 minutes. Whole cell lysates were prepared using RIPA buffer containing protease inhibitor cocktail (Roche, Basel, Switzerland). Protein determination was made using a bicinchoninic acid protein assay (Pierce, Rockford, IL). Clarified lysates were resolved by 4-20% gradient Bis-Tris SDS-PAGE, transferred to nitrocellulose membrane, and probed for antibodies described above. Membranes probed with phospho-antibodies were stripped and reprobed with pan-antibodies and GAPDH as loading control.

Statistical analysis

All statistical analysis was performed using Microsoft Excel. The Student's *t* test was used to evaluate differences observed between experimental groups. Statistical significance was accepted at the 5% level.

References

1. Trott, O. & Olson, A.J. AutoDock Vina: improving the speed and accuracy of docking with a new scoring function, efficient optimization, and multithreading. *J Comput Chem* **31**, 455-61 (2010).
2. Ruiz-Carmona, S. et al. rDock: a fast, versatile and open source program for docking ligands to proteins and nucleic acids. *PLoS Comput Biol* **10**, e1003571 (2014).
3. Andrews, F.H. et al. Dual-activity PI3K-BRD4 inhibitor for the orthogonal inhibition of MYC to block tumor growth and metastasis. *Proc Natl Acad Sci U S A* **114**, E1072-E1080 (2017).
4. Minor, W., Cymborowski, M., Otwinowski, Z. & Chruszcz, M. HKL-3000: the integration of data reduction and structure solution--from diffraction images to an initial model in minutes. *Acta Crystallogr D Biol Crystallogr* **62**, 859-66 (2006).
5. McCoy, A.J. et al. Phaser crystallographic software. *J Appl Crystallogr* **40**, 658-674 (2007).
6. Adams, P.D. et al. PHENIX: a comprehensive Python-based system for macromolecular structure solution. *Acta Crystallogr D Biol Crystallogr* **66**, 213-21 (2010).
7. Emsley, P., Lohkamp, B., Scott, W.G. & Cowtan, K. Features and development of Coot. *Acta Crystallogr D Biol Crystallogr* **66**, 486-501 (2010).
8. Singh, A.R. et al. Single Agent and Synergistic Activity of the "First-in-Class" Dual PI3K/BRD4 Inhibitor SF1126 with Sorafenib in Hepatocellular Carcinoma. *Mol Cancer Ther* **15**, 2553-2562 (2016).

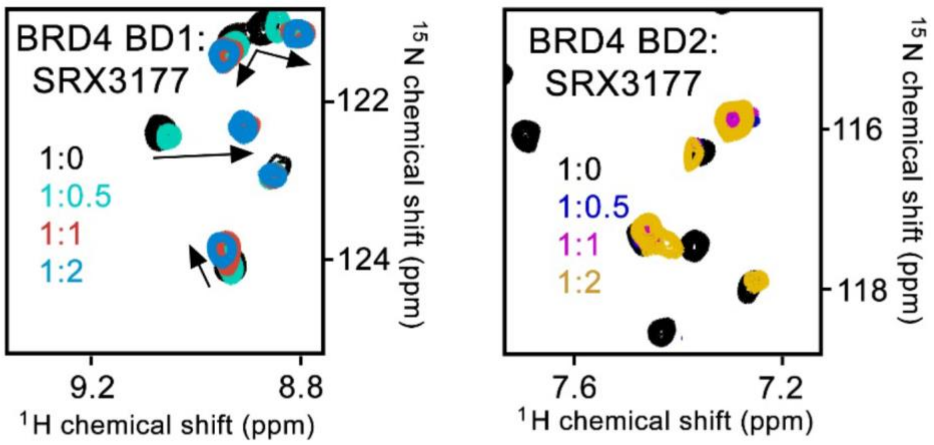

Supplementary Figure S1. Superimposed $_{1}$H,$_{15}$N HSQC spectra of uniformly $_{15}$N-labeled BRD4 BD1, recorded while SRX3177 was titrated in. The spectra are color-coded according to the BD1:SRX3177 molar ratio. Related to Figure 1.

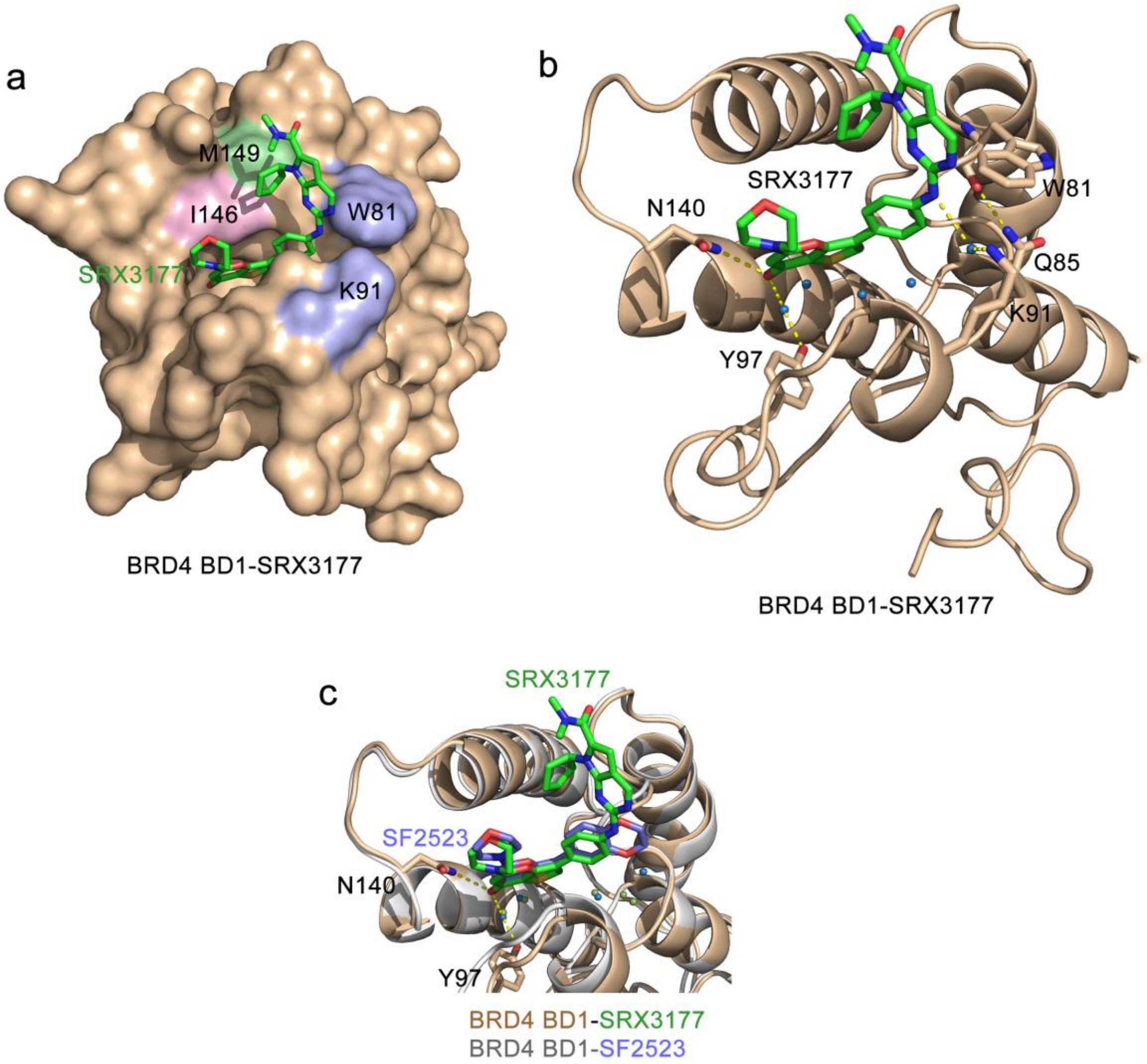

Supplementary Figure S2. BRD4 BD1- SRX3177 structure analysis. (a, b) Surface representation (a) and ribbon diagram (b) of the crystal structure of BRD4 BD1 (light brown) in complex with SRX3177 (green). Water molecules and hydrogen bonds are shown as yellow dashes and blue spheres, respectively. (c) Overlay of the structures of BRD4 BD1 in complex with SRX3177 (green) and SF2523 (blue). Related to Figure 1.

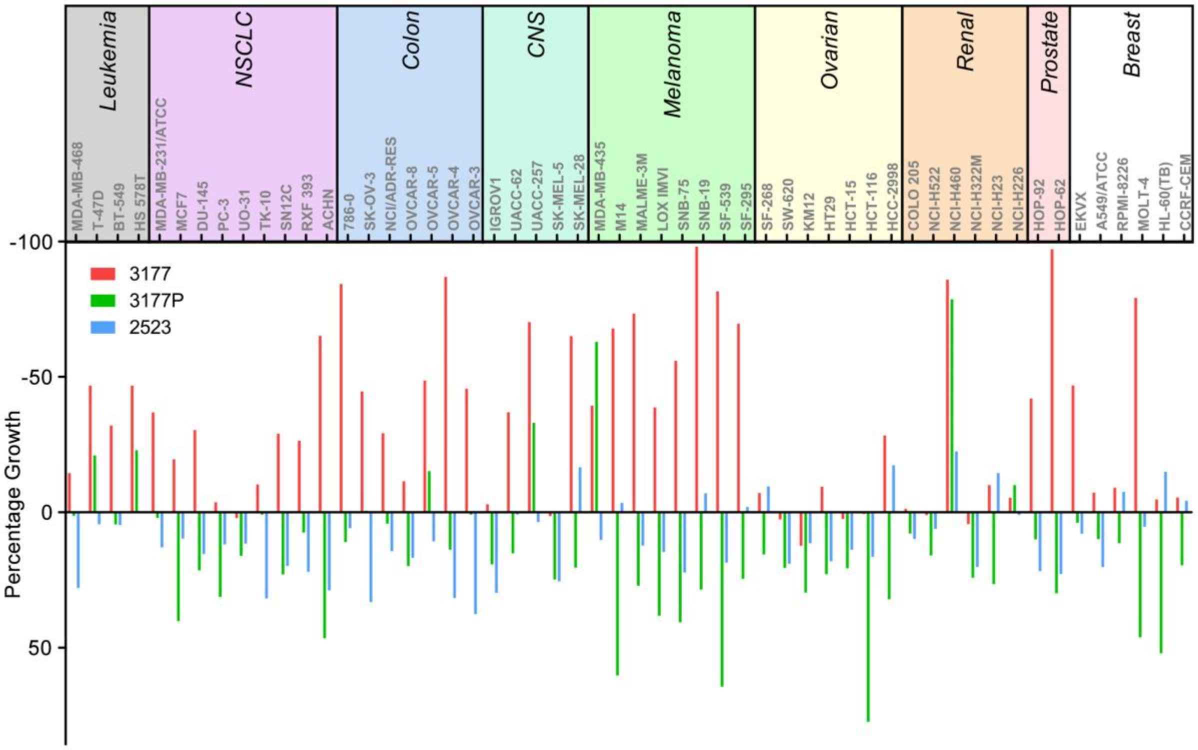

Supplementary Figure S3. SRX3177 has cytotoxic activity throughout cancer cell lines. Percentage of growth or cell death data from the NCI-60 panel was plotted for SRX3177 (red), SRX 3177P, a piperazine derivative of SRX3177 (PI3K dead) (green) and SF2523 (a dual PI3K/BRD4 inhibitor) (blue) and stratified according to cell type and % of growth inhibition (bars values below the X-axis) or cytotoxicity (bars values above the X-axis). Related to Figure 1.

Supplementary Table S2. **Crystallographic X-ray data and refinement statistics**

	BRD4-BD1 in complex with SRX3177
**Data Collection**	
Space group	P 3_1
Wavelength (Å)	1.54
Resolution (Å)	33.15- 2.30 (2.38-2.30)*
Unit-cell dimensions	
a, b, c (Å)	45.318, 45.318, 61.895
a, b, γ (°)	90, 90, 120
No. of measured reflections	19587
No. of unique reflections	6188
Redundancy	3.2 (2.8)
I/σ	12.03 (1.9)
Completeness (%)	98.6 (96.5)
$R_{sym}^{\#}$ (%)	7.5 (48.2)
No. of molecules in ASU	1
Matthews coefficient ($Å^3$ Da^{-1})	2.28
Solvent content (%)	46.1
**Refinement**	
R_{work}/R_{free} (%)	19.27/23.52
No. of atoms	1177
Protein	1094
Ligand/ion	42
Water	41
B-factors ($Å^2$)	57.06
Protein	57.10
Ligand/ion	61.01
Water	51.84
R.M.S.D	
Bond lengths (Å)	0.003
Bond angles (°)	0.90
Ramachandran favored (%)	99.2
Ramachandran allowed (%)	0.78
Ramachandran outliers	0
Rotamer outliers	0
Clashscore	2.24

*Values in parentheses are for the highest resolution shell (Å).

$^{\#}R_{sym} = \sum|I_{obs}-I_{avg}|/I_{avg}$, where I_{obs} is intensity of any given reflection and I_{avg} is the weighted mean I.

Supplementary Table S3. Half maximal inhibitory concentration (IC50) in cell line cytotoxicity assays

Compound	Hepatocellular Carcinoma	Neuroblastoma	Mantle Cell Lymphoma	Tonsillar Epithelia
SRX3177	0.64 µM	0.55 µM	0.83 µM	34 µM
Palbociclib	11.9 µM	9.2 µM	8.4 µM	
Buparlisib + JQ1 + Palbociclib	3.4 µM			1.5 µM

The IC50 values represent the calculated values for curves shown in Figure 1f-j.
